# Advances in the Development of Biotherapeutics

**DOI:** 10.1155/2015/793876

**Published:** 2015-05-20

**Authors:** Pedro H. Oliveira, Juergen Mairhofer, Paula M. Alves, Alvaro R. Lara, Cleo Kontoravdi

**Affiliations:** ^1^Institut Pasteur, Microbial Evolutionary Genomics, Département Génomes et Génétique, Paris, France; ^2^CNRS, UMR3525, Paris, France; ^3^Department of Biotechnology, University of Natural Resources and Life Sciences, Vienna, Austria; ^4^enGenes Biotech GmbH, Vienna, Austria; ^5^Instituto de Biologia Experimental e Tecnológica, Apartado 12, Oeiras, Portugal; ^6^Instituto de Tecnologia Química e Biológica, Universidade Nova de Lisboa, Oeiras, Portugal; ^7^Departamento de Procesos y Tecnología, Universidad Autónoma Metropolitana-Cuajimalpa, Delegación Álvaro Obregón, Mexico; ^8^Centre for Process Systems Engineering, Department of Chemical Engineering, Imperial College London, London SW7 2AZ, UK

Biotherapeutics are currently the fastest growing group of pharmaceuticals, being a treatment option for a variety of chronic and sometimes life-threatening conditions. They represent a diverse group of biological products that broadly encompasses nucleic acids, proteins, whole cells, viral particles, and vaccines. In recent years, great strides have been made towards the development of safer and more effective biotherapeutics, as well as in the adoption of cost-effective, more efficient, and streamlined manufacturing processes. This trend is expected to accelerate in the forthcoming years, as emerging fields related to advanced therapies or personalized medicine mature. Despite these progresses, there are still knowledge gaps across all stages of development, as well as technical and regulatory hurdles that can thwart the approval process of these products.

In this special issue, we are pleased to present a collection of papers covering relevant aspects in the field of biotherapeutics.

The work by T. Lin et al. focuses on cell-penetrating peptides (CPPs). These generally correspond to short basic and amphipathic peptides, with a demonstrated capacity of crossing cell membranes, thereby facilitating the delivery of different types of cargoes such as nucleic acids, peptides, proteins, small molecules, and nanoparticles. The authors describe the use of the J-domain of the Heat Shock Protein 40 to enhance the transduction efficiency of arginine-rich CPPs and suggest the internalization of the latter to take place via macropinocytosis followed by endosomal escape.

Q. Chen and H. Lai review the recent progress in the methodology of agroinfiltration as a solution to overcome the challenge of transgene delivery into plant cells for large-scale manufacturing of recombinant proteins. The authors provide a general overview of gene delivery methodologies in plants, followed by a detailed description of agroinfiltration, its applications and scalability, typical vectors used, and how it can be used in different* Nicotiana* and non-*Nicotiana* hosts.

In the work by F. C. Mariz et al., the development of an intellectual property- (IP-) free platform for human papillomavirus (HPV) 16 L1 protein expression based on the constitutive expression of the PGK1 promoter of the methylotrophic yeast* Pichia pastoris* is reported. The authors also claim to have achieved the intracellular assembly of HPV VLPs in yeast cells for the first time.

S. Fujimaki et al. present us with a thorough review of diabetes mellitus-related changes occurring in the central nervous system and skeletal muscle, particularly in terms of dysfunctional neural stem cells and satellite cells. The authors discuss the beneficial effects of exercise in the prevention and therapy of diabetes, as well as the use of stem cell-based approaches in the context of diabetes treatment.

Due to the complexity of many biologics, the assessment of “equivalence” for products obtained from different manufacturers currently presents a new set of challenges to regulatory agencies, since it is not perfectly clear how similar the biosimilar trastuzumab and its licensed equivalent need to be in terms of physicochemical properties for granting a marketing authorization. To illustrate the complexity of this process C. A. López-Morales et al. used a hierarchical strategy with an orthogonal approach to compare a biosimilar trastuzumab to its reference product in terms of physicochemical and biological attributes.

Finally, J. Zurdo et al. present us with an in-depth review of key aspects that need to be taken into consideration during the development of biotherapeutics, such as those related with the proper definition of a quality by design (QbD) workflow, developability methodologies and risk assessment, and product safety and validation. The authors then illustrate the application of developability tools and strategy described in practical case studies.

Collectively, these papers provide a comprehensive view on the current advances taking place in the broad field of biotherapeutics and on the exciting challenges that lie ahead.

